# Effect of Body Mass Index on Effective Dose in Multi Detector Computed Tomography Abdomen Using Automatic Exposure Control

**DOI:** 10.4314/ejhs.v34i6.9

**Published:** 2024-11

**Authors:** S Shailesh Nayak, Sushil Yadav, Abhimanyu Pradhan

**Affiliations:** 1 Department of Medical Imaging Technology, Manipal College of Health Professions, Manipal Academy of Higher Education, Karnataka, Manipal, 576104, India; 2 Department of Allied Health Sciences, Manipal Tata Medical College, Manipal Academy of Higher Education, Karnataka, Manipal, 576104, India

**Keywords:** Automatic Exposure Control, Body Mass Index, Computed Tomography, Volumetric CT Dose Index, Dose Length Product, kVp, mAs

## Abstract

**Background:**

Computed Tomography (CT) of the abdomen is one of the most frequently performed scans in adults for various abdominal pathologies. Its popularity stems from the immediate image reconstruction following acquisition. However, CT scans are known for their high radiation doses compared to other diagnostic X-ray procedures. This study aimed to analyze the effective dose in patients with varying body habitus during multidetector CT of the abdomen using automatic exposure control.

**Methods:**

This prospective study was conducted in the Department of Radiodiagnosis and Imaging from February 2017 to March 2018. Patients aged 18 and older, regardless of gender, undergoing routine Contrast-Enhanced CT (CECT) of the abdomen were included. Participants were categorized into three groups based on Body Mass Index (BMI): normal weight, overweight, and obese.

**Results:**

A total of 168 patients were enrolled, with a mean age of 49.8 ± 15.6 years, predominantly male (66.1%). Obese individuals exhibited significantly higher effective dose values (16.57 ± 2.27 mSv) compared to normal weight (9.45 ± 0.92 mSv) and overweight individuals (11.88 ± 0.77 mSv) (p < 0.01). Similarly, obese patients had significantly higher values for Computed Tomography Dose Index Volume (CTDIvol) (18.32 ± 2.54 mGy) and Dose Length Product (DLP) (1104.86 ± 151.84 mGycm) compared to normal weight (CTDIvol: 11.38 ± 1.24 mGy; DLP: 630.55 ± 61.57 mGycm) and overweight individuals (CTDIvol: 13.56 ±1.15 mGy; DLP: 792.37 ± 51.56 mGy*cm) (p < 0.05).

**Conclusion:**

The effective dose received by obese patients during abdominal CT exams with Automatic Exposure Control (AEC) is nearly double that of normal-weight patients.

## Introduction

The use of Computed Tomography (CT) has increased significantly over the past two decades, making it one of the most popular imaging modalities in medical diagnostics. CT of the abdomen is frequently performed for various abdominal pathologies. Its rapid scan times facilitate immediate diagnosis and prompt treatment for patients (1). CT employs X-ray radiation, which passes through the patient and is detected by specialized electronic sensors. Medical imaging is the primary source of ionizing radiation exposure from artificial sources. The As Low As Reasonably Achievable (ALARA) principle governs the use of ionizing radiation in medical imaging, emphasizing that diagnostic evidence should be obtained at the lowest possible dose (3).

There are growing concerns about the radiation exposure patients face during CT studies. Although CT accounts for only about 10% of X-ray-based exams, it contributes to nearly 50% of the total radiation dose associated with medical imaging (4). The annual dose from background radiation is approximately 3 mSv, while a chest radiograph delivers about 0.02 mSv, and a CT scan of the abdomen and pelvis delivers around 10 mSv. The rapid increase in CT availability and the introduction of multidetector row CT (MDCT) have led to higher patient volumes and expanded clinical use (4, 5).

Recent studies have assessed the risk of developing cancer from diagnostic X-ray procedures. According to Gonzalez and Darby (6), the estimated cancer risk associated with all diagnostic X-ray procedures ranges from 0.6% to 3.2% in developed countries. Since the data for these estimates were collected between 1991 and 1996, the risks today are likely higher. Consequently, the radiation doses from CT exams may approach or exceed those that raise the risk of developing cancer, contributing to lifetime cancer mortality risk compared to background rates (7).

The effective dose (ED) is crucial for determining cancer risk from CT. ED is a weighted sum of all irradiated organ doses, reflecting the varying radio sensitivities of body organs. It is an important dose descriptor in CT related to stochastic effects in patients (8). The ED from a specific CT scan is influenced by factors such as the CT scanner design, patient size, tube current, and scanning time-milliampere-seconds(mAs). A patient's weight is significant since thicker tissues produce more scatter and attenuate more X-rays (3). Higher tube currents are needed to counteract this process, improving image quality but increasing radiation dose to the patient and reducing noise.

The automatic exposure control (AEC) system is one strategy recommended to reduce patient radiation doses during CT imaging. AEC significantly lowers radiation exposure while maintaining or enhancing image quality. However, overweight patients may receive disproportionately high radiation doses when AEC systems are employed, as imaging obese patients is often complicated by increased image noise at lower radiation doses (9, 10, 11).

In MDCT scans, the dose received by the patient also depends on scan length and the patient's anterior-posterior and transverse diameters. However, radiation dose is influenced by mass or BMI. BMI is a better measure for estimating patient size than weight alone, as it considers height and body composition. The current study aims to analyze the influence of BMI on effective dose (ED) in abdominal CT exams using AEC in MDCT.

## Methods

**Study design**: This prospective study was conducted among patients undergoing Contrast-Enhanced CT of the abdomen in the Department of Radiodiagnosis from February 2017 to March 2018. Patients aged 18 and older, regardless of gender, were included. Individuals with a history of trauma or non-cooperative patients were excluded. Informed consent was obtained from all participants. Patient demographic and medical data were gathered from hospital records. Patients were categorized into three groups based on BMI: normal weight, overweight, and obese, with 56 patients in each group.

All CT scans were performed on a 64-slice CT (Philips Brilliance MDCT) using a conventional protocol with 120 kVp and 250 mAs. Technical parameters included a 5 mm slice thickness, 5 mm increment, 64 x 0.625 mm detector width, 350 mm field of view (FOV), 512 x 512 matrix size, 0.98 pitch, and a 0.75-second rotation time. An 80 ml dose of contrast media (Iohexol 300 mg I/ml, GE Healthcare) was injected using a dual-head pressure injector (MEDRAD, Stellant) with images acquired after a post-threshold delay of 13 seconds.

Dose information, including Dose Length Product (DLP) and Computed Tomography Dose Index volume (CTDIvol), was recorded from the CT monitor after each scan. The effective dose (ED) was calculated from DLP using the formula:

E=k×DLPE = k\times DLPE=k×DLP, where kkk is the conversion factor specific to the anatomical region. The kkk value for CT abdomen is 0.015 (12).

**Statistical analysis**: Quantitative data were presented as Mean ± SD, while qualitative data were expressed as proportions and percentages. Microsoft Excel and the Statistical Package for Social Sciences (SPSS) version 25 were used for data cleaning and analysis. Relationships were examined using the Mann-Whitney U test and the Jonckheere-Terpstra test. A significance level of 5% was applied.

**Study ethics**: The study was approved by the Institutional Ethical Committee (IEC47/2017).

## Results

**Demographics**: A total of 168 patients undergoing routine abdomen CT scans were enrolled. The mean age of participants was 49.82 ± 15.67 years, with most being male (68%). Patients were divided equally into three groups: normal weight, overweight, and obese. The overall mean BMI was 26.08 ± 4.52, with mean BMI values of 20.67 ± 1.61 for normal weight, 26.29 ± 1.05 for overweight, and 31.30 ± 0.98 kg/m^2^ for obese patients (Table 1).

**Table 1:** Demographics

Variables	Frequency (%)
Average Age	49.82±15.67
**Gender:**	
Male	111 (66.1)
Female	57 (33.9)
BMI (kg/m2)	26.08±4.52
**BMI Category:**	
Normal weight (kg/m^2^)	20.67±1.61
Overweight (kg/m2)	26.29±1.05
Obese (kg/m2)	31.30±0.98

BMI: Body Mass Index

**Comparison of CT data with demographics**: The mean ± SD values for CT Dose Index Volume (CTDIvol), Dose Length Product (DLP), and scan length (FOV) were 14.42 ± 3.39 mGy, 842.59 ± 220.69 mGy*cm, 508.7 ± 36.36, and 446.04 ± 44.99, respectively (Table 2). No significant differences in gender were observed across the BMI categories.

**Table 2:** CT Related data

Variable	Mean±SD
CTDIvol (mGy)	14.42±3.39
DLP (mGy*cm)	842.59±220.69
Scan length	508.7±36.36
FOV	446.04±44.99
ED (mSv)	12.63±3.31

CTDIvol: CT dose index volume; DLP: Dose Length Product; ED: Effective Dose; FOV: Field of View

**Figure 1:**
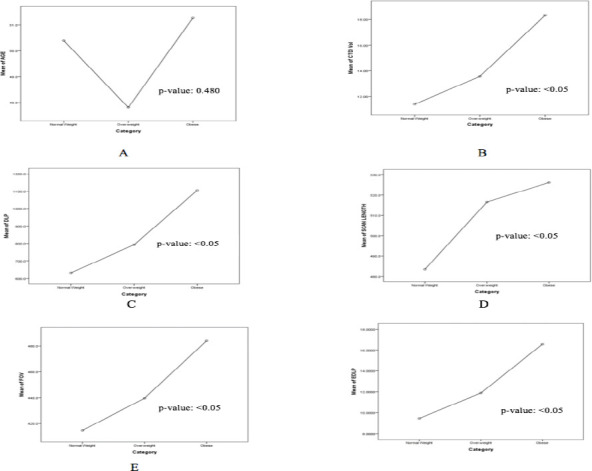
CT Data and BMI Category: A: Relation of Age and BMI; B: Relation of CTDIVol and BMI; C: Relation of DLP and BMI; D: Relation of Scan Length and BMI; E: Relation of FOV and BMI; F: Relation of EDLP and BMI

Significant differences were noted in CT data relative to BMI. Post-hoc analysis revealed significantly higher values for CTDIvol, DLP, scan length, FOV, and ED in obese individuals compared to those with normal and overweight BMI (Table 3 & Figure 1).

**Table 3:** Comparison of CT data with BMI

Variables	BMI Category			p-value
		
	Normal weight (kg/m2)	Overweight (kg/m2)	Obese (kg/m2)	
Age	50.39±16.64	47.80±14.63	51.26±15.73	0.480
CTDIvol (mGy)	11.38±1.24	13.56±1.15	18.32±2.54	0.02
DLP (mGy*cm)	630.55±61.57	792.37±51.56	1104.86±151.84	0.015
Scan Length	483.39±35.80	516.60±28.96	526.25±29.41	0.04
FOV	414.58±37.68	439.55±35.53	484.0±30.79	0.005
ED (mSv)	9.45±0.92	11.88±0.77	16.57±2.27	0.01

CTDIvol: CT dose index volume; DLP: Dose Length Product; ED: Effective Dose; FOV: Field of View

## Discussion

The radiology community has recently taken patient radiation exposure and methods to reduce doses more seriously (13). This study evaluates the correlation between Body Mass Index (BMI) and the Effective Dose (ED) administered during abdominal examinations utilizing Automatic Exposure Control (AEC) in the context of Multidetector Computed Tomography (MDCT). A total of 168 patients who underwent routine abdomen CT scans were enrolled. Participants were divided into three groups based on their BMI: normal weight, overweight, and obese.

Most of the recruited participants were male (66.1%), with a mean age of 49.82 ± 15 years. The overall mean BMI among study participants was 26.08 ± 4.52. Additionally, the mean BMI in the normal, overweight, and obese groups was 20.67 ± 1.61, 26.29 ± 1.05, and 31.30 ± 0.98, respectively.

The current study reported mean ± SD values for the CT Dose Index Volume (CTDIvol), Dose Length Product (DLP), scan length, and field of view (FOV) of 14.42 ± 3.39, 842.59 ± 220.69, 508.7 ± 36.36, and 446.04 ± 44.99, respectively. Compared to studies conducted by Bashier et al. (14) and Amer et al. (15), these results were generally lower. Bashier et al. reported a CTDIvol of 16.02 ± 8.85, while Amer et al. found a CTDIvol of 12.38 ±7.83.

In this study, we primarily evaluated CT scan data about patient gender. However, our analysis indicated that gender did not show a significant difference across the BMI categories.

AEC has become more commonly used in CT in recent years to minimize radiation exposure. Understanding how radiation exposure and effective dose vary with AEC is essential. Numerous studies have evaluated the effects of AEC on effective doses for various examinations worldwide (16).

Our study analyzed the correlation between effective dose and BMI using AEC. The results showed a significant variation in effective doses among the three BMI categories, indicating that the risk associated with radiation exposure is primarily due to differences in patient BMI. This variation stems from the amount of radiation exposure used during scans of patients with different BMIs, leading to differences in organ doses. A similar observation was made by Schindera et al. (17), who noted that abdominal organ doses for larger patients increased by up to 528% compared to smaller patients. However, a study by Meeson et al. (18) reported that mass and BMI are not suitable measures for estimating patient dose relative to patient size, as the distribution of subcutaneous and intra-abdominal fat plays a significant role in the effective dose received.

Post-hoc analysis revealed significantly higher values for CTDIvol, DLP, scan length, FOV, and ED in obese individuals compared to those with normal and overweight BMI. This finding aligns with Birgani et al. (19), who demonstrated that effective doses increase with rising BMI. Similar results were reported in a recent study by Sebelego et al. (20), which indicated that CTDIvol and DLP were higher among obese patients than normal-weight patients.

The current study has certain limitations. One limitation pertains to the recommendations for dose efficiency with AEC across diverse BMI levels, suggesting that operators tailor image quality settings to match patient body habitus. Additionally, considering the cross-sectional area of the scanned body may provide a more reliable measure of patient size. This investigation highlights the need for further research focused on optimizing protocols for specific BMI levels and comparing subjective image quality against standard protocols in a larger cohort.

Future studies could involve a greater number of subjects, covering different anatomical areas and scanners from various vendors, including pediatric patients, who are more sensitive to radiation. While this study focused on abdominal scans, including patients with other anatomical regions, such as the chest, could provide additional insights, as CTDIvol may vary with patient BMI. For this study, we utilized patient BMI as a straightforward measure of size, emphasizing the need for future dose optimization studies to determine the most appropriate scan parameters based on patient size.

In conclusion, this study emphasizes the significant impact of BMI on radiation exposure during MDCT abdominal imaging when employing automatic exposure control. The results revealed that individuals with higher BMI (obese) demonstrated significantly elevated values for CTDIvol, DLP, scan length, FOV, and ED compared to individuals with normal or overweight BMI levels. This underscores the importance of tailoring imaging protocols and dose optimization strategies to accommodate patients with varying BMI levels, ensuring diagnostic quality and radiation safety in clinical practice. Further research may be warranted to refine protocols and explore additional avenues for minimizing radiation exposure in this patient population.
